# The Cooperation of *Bifidobacterium longum* and Active Vitamin D3 on Innate Immunity in *Salmonella* Colitis Mice via Vitamin D Receptor

**DOI:** 10.3390/microorganisms9091804

**Published:** 2021-08-25

**Authors:** Fu-Chen Huang, Shun-Chen Huang

**Affiliations:** 1 Department of Pediatrics, Kaohsiung Chang Gung Memorial Hospital and Chang Gung University College of Medicine, Kaohsiung 833, Taiwan; 2 Department of Pathology, Kaohsiung Chang Gung Memorial Hospital, Kaohsiung 833, Taiwan; shuang@cgmh.org.tw

**Keywords:** *Salmonella* colitis, probiotic, vitamin D, inflammation, antimicrobial peptide

## Abstract

*Salmonella* spp. remains a major public health problem for the whole world. Intestinal epithelial cells serve as an essential component of the mucosal innate immune system to defend against *Salmonella* infection. Our in vitro studies showed probiotics and active vitamin D have similar effects on innate immunity in *Salmonella*-infected intestinal epithelial cells, including antimicrobial peptide and inflammatory responses, to protect the host against infection while downregulating detrimental overwhelming inflammation. Hence, we investigated the synergistic effects of probiotics and active vitamin D on *Salmonella colitis* and translocation to liver and spleen by in vitro and in vivo studies. The *Salmonella* colitis model is conducted with 6–8 *w*/*o* male C57BL/6 mice: Streptomycin (20 mg/mouse p.o.)-pretreated C57BL/6 mice are mock infected with sterile PBS or infected orally with 1 × 10^8^ CFU of a S. Typhimurium wild-type strain SL1344 for 48 h. The mice in the treated groups received 1, 25D daily (0.2 ug/25 g/d) and/or 1 × 10^8^ CFU of probiotics, *Lactobacillus rhamnosus GG* (LGG) and *Bifidobacterium longum* (BL) by intragastric administration for 14 days. The in vivo study demonstrated the combination of probiotic *Bifidobacterium longum* and active vitamin D3 had the synergistic effects on reducing the severity of *Salmonella* colitis and body weight loss in C57BL/6 mice by reducing cecal inflammatory mIL-6, mIL-8, mTNF-α and mIL-1β mRNA responses, blocking the translocation of bacteria while enhancing the antimicrobial peptide mhBD-3 mRNA in comparison to the infection only group. However, LGG did not have the same synergistic effects. It suggests the synergistic effects of *Bifidobacterium longum* and active vitamin D on the antibacterial and anti-inflammatory responses in *Salmonella* colitis. Therefore, our in vivo studies demonstrated that the combination of probiotic *Bifidobacterium longum* and active vitamin D3 has the synergistic effects on reducing the severity of *Salmonella* colitis via the suppression of inflammatory responses, and blocking the translocation of bacteria through the enhancement of antimicrobial peptides.

## 1. Introduction

*Salmonella* spp. are important Gram-negative pathogens of humans known to cause a wide variety of diseases ranging from mild diarrhea to severe systemic complications. *Salmonella* spp. remain a major public health problem for the whole world [1]. *Salmonella* infection is even more detrimental in the developing world. This century, it has been noticed the incidence of *Salmonellosis* in humans caused by S. enteritidis or multi-drug-resistant strains of *S. typhimurium* has increased [2] with similar problems encountered globally [3,4,5,6]. A 5-year prospective study of antibiotic resistance among children hospitalized for acute diarrhea with *Salmonella enterica* showed high prevalence of resistant strains [7]. In addition, excess mortality was associated with antimicrobial drug-resistant *S. typhimurium* [8]. Better intervention strategies are needed to reduce the overuse of antimicrobial agents and decrease drug-resistant *Salmonellosis*.

Following oral infection, the intestinal mucosa serve not only as a barrier to bacteria invasion into the gut, but also integrate the innate immunity of the host by the production of inflammatory cytokines, chemokines, and antimicrobial peptides. Antimicrobial peptides (AMPs), e.g., human β–defensin-2 (hBD-2), are crucial for host defense against the invasion of *Salmonella*, while chemokines, e.g., IL-8, recruit neutrophils from the circulation into the subepithelial tissue to cause colitis.

It has been found that the induction of antimicrobial peptide genes’ expression by vitamin D [9] explores the ‘antibiotic’ effect of vitamin D. Increasing research projects have unraveled the important role of vitamin D in the regulation of innate and adaptive immunity [10,11,12]. 1,25-dihydroxyvitamin D3 (1,25D3), the active form of vitamin D, induced the expression of two AMPs, hBD-2 and cathelicidin [13,14], in several cultured cells in response to bacterial pathogens. Lower levels of vitamin D were associated with severe illness in sepsis patients [15]. 1,25D3 can significantly enhance the production of AMPs levels to prevent urinary tract infections [16] or reduce the use of antibiotics in patients with frequent respiratory tract infections [17]. Giving vitamin D supplementations to children who had low blood levels of vitamin D decreased their risk of respiratory infections [18]. Thus, supplementation with vitamin D3 could be a new strategy in reducing antibiotic use and may indirectly prevent the development of bacterial resistance. 1,25D3 supplementation was effective in ameliorating acute or chronic colitis, including human and animal studies [19,20]. We recently demonstrated that active vitamin D reduces the severity of *Salmonella* colitis and invasion in mice by anti-inflammatory and antibacterial effects [21]. Our previous in vitro studies [22,23] also showed that active vitamin D enhances hBD-2 expression in *Salmonella*-infected intestinal epithelial cells (IECs), so that the host is protected against infection. In addition, the active vitamin D downregulates proinflammatory responses (IL-8 & IL-1β) via the vitamin D receptor (VDR); consequently, the host may escape the detrimental effects of overwhelming inflammation.

Probiotic bacteria are microorganisms that benefit the host through the improvement of the balance of the intestinal microflora and possibly by the augmentation of host defense systems. Probiotics have been shown to have antibacterial activity against *Escherichia coli* (*E. coli*) [24]. Clinically, probiotics can be used as an add-on therapy in acute gastroenteritis in children by shortening the duration of diarrhea [25] and hospital stay, resulting in both social and economic benefits. However, the beneficial effect in viral gastroenteritis is not observed in bacterial colitis, though we observed that probiotics could downregulate IL-8 and IL-1β expression in *Salmonella*-infected IECs [26,27].

Therefore, we investigated the cooperative effect of probiotics and active vitamin D on the severity of *Salmonella* colitis and invasion in mice by regulating antimicrobial peptides and anti-inflammatory responses.

## 2. Materials and Methods

### 2.1. Bacterial Strains

The naturally streptomycin-resistant *S. enterica* serovar Typhimurium (S.Tm) wild-type strain SL1344, *Lactobacillus rhamnosus* GG (LGG) (ATCC 53103) and *Bifidobacterium longum* (BL) (ATCC15707) were provided by Food Industry Research and Development Institute (FIRDI, Taipei City 10015, Taiwan).

The naturally streptomycin-resistant S.Tm wild-type strain SL1344, *LGG* and *BL* were grown as per manufacturer’s description. The SL1344 were collected using centrifugation at 14,000× *g* for 5 min, washed with sterile phosphate-buffered saline (PBS), and resuspended without antibiotics at a density of 4 × 10^8^ CFU/mL. Solutions of *LGG* and *BL* were diluted 1:100 in fresh MRS broth and sub-cultured for 16 h at 37 °C under an anaerobic atmosphere, washed and resuspended to final concentration 10^8^ CFU/mL.

### 2.2. 1,25(OH)2D3 Treatment

1,25D3 was dissolved in corn oil to the final concentration of 30 mg/mL, and the mice were treated via oral gavage with active vitamin D3 (1,25D3) (0.2 μg/25 g mice) daily. The control mice were fed with the corresponding amount of ethanol diluted in corn oil.

### 2.3. Animal Experiments

All mice were generously provided by National Laboratory Animal Center. Forty-two six to eight week old female C57BL/6 mice specific-pathogen–free mice were fed in SPF room in Kaohsiung Chang Gung Memorial Hospital animal center. Animal experiments were approved by the Kaohsiung Chang Gung Memorial Hospital Institutional Animal Care and Use Committee according to the legal requirements (Approval No. 2016122117, 21 February 2017). For the experiments, the mice were housed in individually ventilated cages and transferred to negative pressure room. Mice were divided into seven groups: NA (Open control); VD (vitamin D3 only); *S.*Tm (Comparison group); LGG (LGG only); VD+LGG (vitamin D3 and LGG); BL (BL only); VD+BL (vitamin D3 and BL).

Before induction of the colitis, mice were oral gavage given vitamin D3 0.2 μg/25 g mice (VD group) or 10^8^ CFU probiotics (LGG and BL group) or both vitamin D3 and probiotics (VD+LGG and VD+BL group) for 4 days. The other groups were fed 100 μL sterile water (Open control) or 100 μL PBS (*S.*Tm group).

Water and food were withdrawn 3 h before treatment with 20 mg streptomycin (100 μL sterile water for open control) per oral gavage. Then, animals were supplied with water and food at will. Then, 24 h after streptomycin treatment, food and water were withdrawn again for 3 h and infected with *S. enterica* serovar Typhimurium SL1344 10^8^ CFU (suspended in 100 μL PBS) or treated with sterile water (100 μL sterile water for open control). The food and water were supplied at will again. Then, 48 h after infection, the mice were again treated with vitamin D3 0.2 μg/25 g mice (VD group) or 10^8^ CFU probiotics (LGG and BL group) or both vitamin D3 and probiotics (VD+LGG and VD+BL group) for 7 days. Other groups were fed 100 μL sterile water (Open control) or 100 μL PBS (S.Tm group). On day 14, we collected the submandibular bleeding by using lancets. Then, mice were sacrificed by CO2 narcosis and their intestinal tracts, spleen, and liver were removed, weighed, flushed with ice-cold PBS, and cut. Tissues were treated for further analysis.

### 2.4. Analysis of S.Tm Loads in the Spleens and Livers

All the tissues removed from mice were removed aseptically, weighed and recorded. To analyze the colonization of bacteria, the liver and spleen were homogenized in 4 °C cold PBS with 1% triton X-100 using a Potter homogenizer; using a micropestle to mince the tissue as much as possible. To determine the numbers of *S*.Tm colonized, the numbers of CFU were determined by plating appropriate dilutions on MacConkey agar plates (with 50 μg/mL streptomycin) cultured for 16 h at 37 °C under mild aeration. The minimal detectable values were 20 CFU/organ in the spleen and 100 CFU/organ in the liver.

### 2.5. Histological Colitis Scoring

Postmortem, the entire colon was removed, and the colon length and weight were measured. Segments of the cecum and colon were fixed and embedded in paraffin according to the standard procedures. Alternatively, tissue samples were embedded, snap-frozen in liquid nitrogen, and stored at −80 °C. Part of the cecum was harvested, fixed in 10% formalin, processed, and embedded in paraffin according to the standard protocol. Cryosections (5 μm) were mounted on glass slides, air-dried for 2 h at room temperature, and stained with hematoxylin and eosin (H & E). Histological scoring was performed in a blinded fashion by a trained pathologist, with a combined score for submucosal edema (score, 0–3), epithelial integrity (score, 0–3), number of goblet cells (score, 0–3) and polymorphonuclear leukocytes in the lamina propria (score, 0–4) [28]. The combined pathological score for each tissue sample was determined as the sum of these scores; it ranges between 0 and 13 arbitrary units and indicates the levels of inflammation in the cecum.

The diarrhea situation was scored as follows: 5 = Mice alive and energetic; 4 = Mice experiencing diarrhea and pasty stools; 3 = Mice experiencing loose stools and reduced mobility; 2 = Mice alive but weak and experiencing abnormal behavior; 1 = Mice lost their lives.

### 2.6. Quantitative Real-Time PCR Analysis of Cecum RNA

Samples of the cecum were obtained, immediately snap-frozen in liquid nitrogen, and stored at −80 °C. Total RNA was extracted from the cecum tissue or infected cultured cells, using TRI Reagent (Ambio #15596018, Pittsburgh, PA, USA) and Directzol RNA MiniPrep kit (Zymo Research, Irvine, CA, USA), according to the manufacturer’s instructions. The RNA was reverse-transcribed into cDNA by using PrimeScript^TM^ RT reagent Kit (TaKaRaCat #RR037A, San Jose, CA, USA) in a 20 μL reaction volume with a final concentration of 1 μg total RNA. Then, cDNA samples were subjected to quantitative real time PCR using ABI 7500 Real-Time PCR System (Applied Biosystem) and FAST SYBR GREEN MASTER MIX (Thermo Fisher Scientific, Waltham, MA, USA)according to the manufacturer’s directions.

Primers (Genomics, New Taipei City, Taiwan) for the genes that we interested in and internal reference were as follows: Beta-actin (β-actin): forward, 5′-TGT CGA GTC GCG TCC ACC-3′, reverse, 5′-TCG TCA TCC ATG GCG AAC TGG-3′; VDR: forward, 5′-ACCCTGGTGACTTTGACCG-3′, reverse, 5′-GGCAATCTCCATTGAAGGGG-3′; Chemokine (C-X-C motif) ligand 2(CXCL2): forward, 5′-GCCCAGACAGAAGTCATAGCC-3′, reverse, 5′-GCTCCTCCTTTCCAGGTCAG-3′; Mouse beta defensin-3 (mBD-3, analog of hBD-2): forward, 5′-GCATTGGCAACACTCGTCAGA-3′, reverse, 5′-CGGGATCTTGGTCTTCTCTA-3′; Interleukin 1 beta (IL-1β): forward, 5′-AGCTTCCTTGTGCAAGTGTC-3, reverse, 5′-TTGGGGTCCGTCAACTTCAA-3′; Tumor Necrosis Factor-Alpha (TNFα): 5′-CTCCAGGCGGTGCCTATGTC-3′, reverse, 5′-CCATTTGGGAACTTCTCATCCCTTT-3′; Interleukin 6 (IL-6): forward, 5′-GTTCCTCTCTGCAAGAGACTTC-3′, reverse, 5′-AGTCTCCTCTCCGGACTTGT-3′.

We used the ABI 7500 Real-Time PCR System (Applied Biosystem) to set up the reactions. The reaction protocol was set as below. The denaturation step was set up at 95 °C for 1 min, primer annealing step was set at 54 °C for 1 min and the extension step was set at 72 °C for 2 min. These three steps were repeated for 25–35 cycles. The threshold cycle (Ct), which represented the duplication cycle number, was determined by the fluorescent value in the exponential phase of the amplification. The ABI7500 software (SDS V2.3) was used to obtain raw fluorescence data (Rn and DRn) for analysis. The relative number of transcripts was normalized to the amount of β-actin transcript by subtracting the mean Ct value of the latter from the mean Ct value of the former for each experimental condition. The difference between the normalized Ct values of the infected cells and the control cells is a measure of the change in mRNA expression. Many aspects of the MIQE guidelines were taken into consideration for the methods and analysis [29].

### 2.7. RNA Interference (RNAi) in Cultured Cells

RNAi experiments in cultured cells were performed as described previously [30,31,32]. Briefly, cultured SW480 cells were transfected with VDR siRNA according to the manufacturer’s protocol to knockdown VDR expression. After transfection, the cultured cells were infected by SL1344 with or without treatment of vitamin D or BL. Then, total RNA was extracted from the cells and number analyzed for mRNA expression.

### 2.8. Statistical Analysis

All the above experiments were performed in triplicate with similar results. We made use of GraphPad Prism 8 software (GraphPad Software, San Diego, CA, USA) to performed the statistical analysis. For three or more nonparametric variables, we used Kruskal–Wallis one-way ANOVA to determine the variance. A *p*-value of <0.05 was considered statistically significant.

## 3. Results

### 3.1. In Vivo Study

#### 3.1.1. Combination of Bifidobacterium Longum and Active 1,25D3 Attenuates the Severity of Salmonella Colitis in Mice

**Figure 1:**
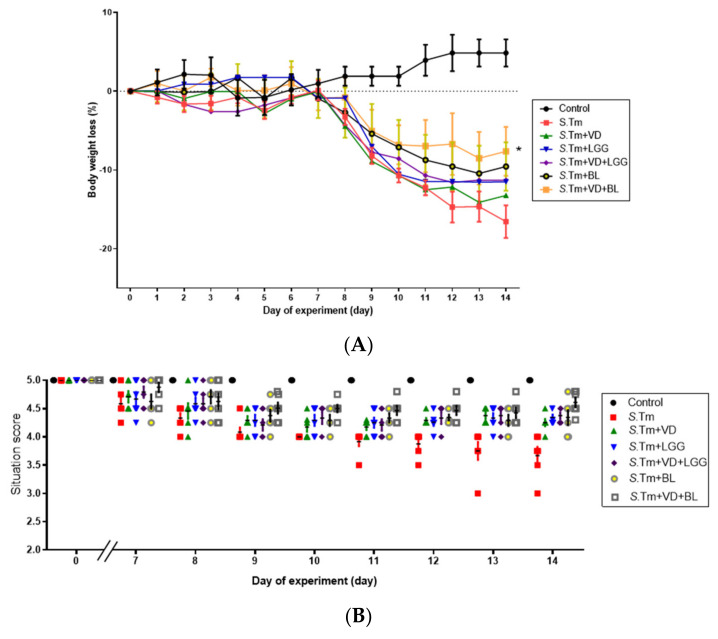

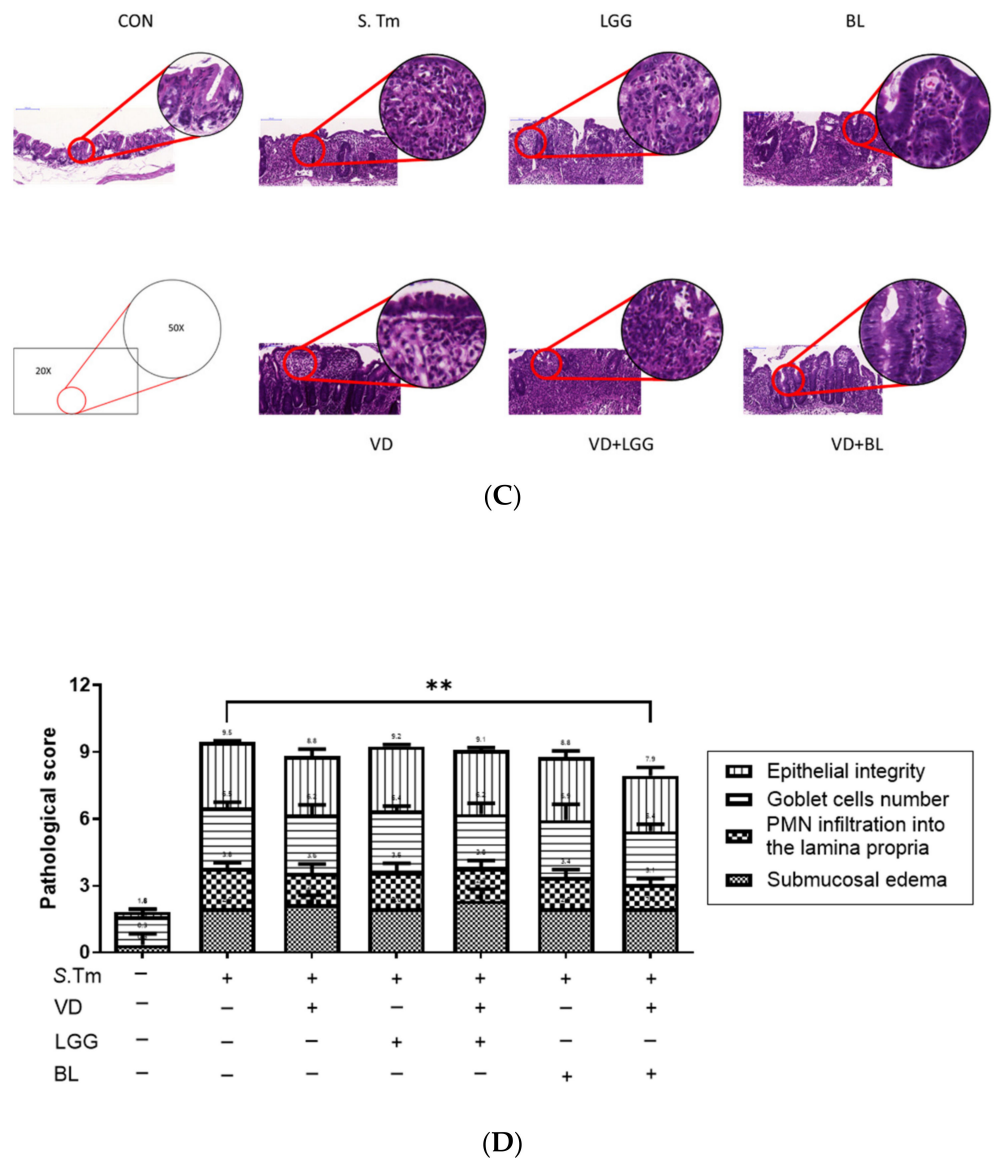
Combination of probiotics and active 1, 25 D3 attenuates the severity of *Salmonella* colitis in mice. We orally infected 6−8 week-old female C57BL/6 mice with 10^8^ CFU of SL1344 (*S*.Tm). Before and after infection, mice were gavaged with vehicle control (5% dimethyl sulfoxide), 1,25 D3 (VD), *Lactobacillus rhamnosus* GG (LGG) or *Bifidobacterium longum (BL),* daily as shown above. Loss of body weight (**A**) and scores of diarrhea situation (**B**) in mice were recoded daily. Cecum was resected and fixed in formaldehyde, and sections were stained with hematoxylin and eosin stain. (**C**) Representative histological images (× 20 and × 50 magnification) of cecum from the different experimental groups. (**D**) The pathological scores for colitis at the cecum of mice. The data shown are means ± SEM (*n* = 6 mice/group). ** *p* < 0.01.

To study the effects of combined 1,25D3 and probiotics on the severity of *Salmonella* colitis, we investigated the cecal pathology of infected WT mice in the presence or absence of 1,25D3 or probiotics treatment. Consistent with our previous study [21], we observed obvious pathological changes in the H&E-stained cecum sections from the infected WT mice 48 h post-infection in Figure 1C. In contrast, the combination of BL and VD significantly attenuates the severity of *Salmonella* colitis from the histopathological analysis of cecum. Using the histological scoring, the severity of *Salmonella* colitis was decreased more significantly in the BL-VD combination-treated groups than in the infection-only WT mice (Figure 1D).

In Figure 1, we observed that the combination of BL and 1,25D3 synergistically reduced the severity of *Salmonella* colitis, including body weight loss, situation of conditions and pathologic scores, in C57BL/6 mice, but LGG had no significantly synergistic effect.

#### 3.1.2. Combination of 1,25D3 and *Bifidobacterium longum* Exerts Cecal Anti-Inflammatory Responses and Antimicrobial Peptide in Salmonella Colitis Mice

**Figure 2:**
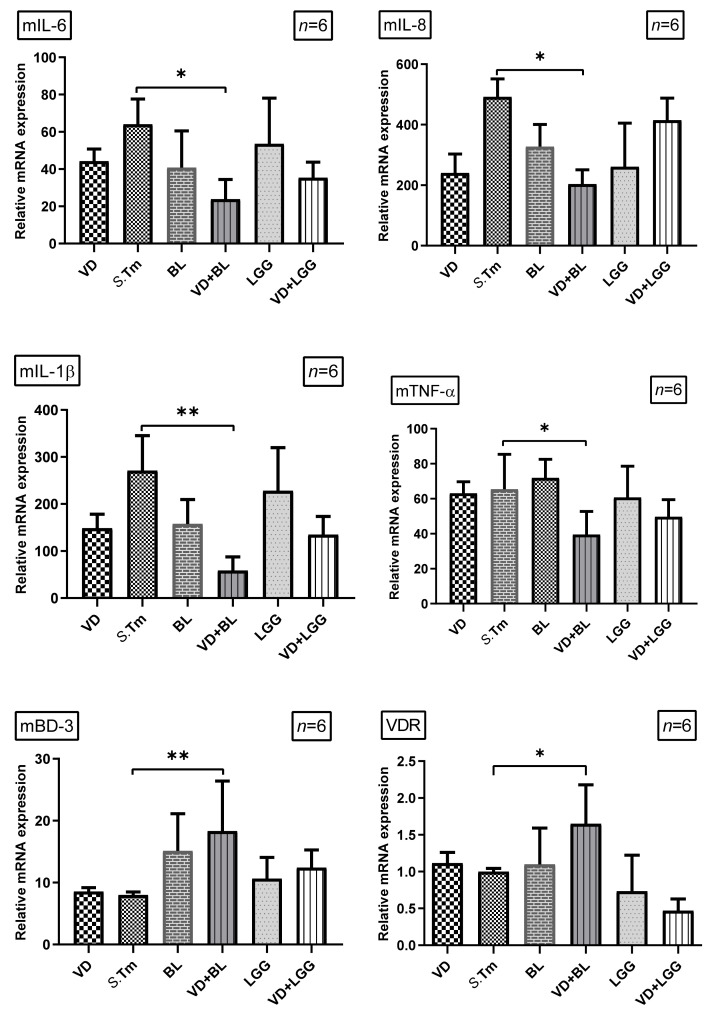
The immunoregulatory effects of combined 1,25D3 and *Bifidobacterium Longum* on cecal cytokines and antimicrobial peptide in *Salmonella* colitis mice. Before and after infection, mice were gavaged with vehicle control (5% dimethyl sulfoxide), 1,25D3 (VD), *Lactobacillus rhamnosus* GG (LGG) or *Bifidobacterium longum* (BL), or combination of VD and BL (VD+BL) or LGG (VD + LGG). At the end of the experiment, total RNA was extracted from the cecal tissues. mIL-6 mIL-8, mIL-1β, mTNF-α, mBD-3 and VDR mRNA expressions were analyzed using real-time quantitative PCR. Values were measured as fold increase compared to the level of control group. The data shown are means ± the SEM (*n* = 6 mice/group). An asterisk indicates the significant differences compared to *Salmonella* infection only group, based on one-way ANOVA. * *p* < 0.05, ** *p*< 0.01.

To investigate the effects of combination of *Bifidobacterium logum* and 1,25D3 on the inflammatory and antimicrobial peptide responses in *Salmonella*-infected mice, the gene expression of cytokines and antimicrobial peptide was quantified using real-time PCR in the cecal tissue of *Salmonella*-infected mice with or without treatment of 1,25D3 or probiotics. Cecal gene expression of mIL-6, mIL-8, mIL-1β and mTNF-α, and mBD-3 (Figure 2) were significantly increased in *Salmonella*-infected mice. In contrast, mIL-8, mIL-1β and mTNF-α were synergistically suppressed in the cecal tissue of *Salmonella* colitis mice treated with a combination of 1,25D3 and *Bifidobacterium longum*, whereas mBD-3 was synergistically enhanced.

We observed that a combination of BL and 1,25D3 reduced cecal mIL-6 (23.87 ± 7.06 vs. 64.00 ± 21.93, *p* < 0.05), mIL-8 (203.50 ± 53.74 vs. 491.70 ± 59.72, *p* < 0.005), mIL-1beta (58.21 ± 12.01 vs. 271.00 ± 21.96, *p* < 0.01) and mTNF-alpha (42.82 ± 7.77 vs. 71.88 ± 14.72, *p* < 0.005) but increased mBD-3 mRNA (18.32 ± 3.30 vs. 8.00 ± 0.47, *p* < 0.005) expressions. Furthermore, the combination of BL and 1,25D3 increased VDR mRNA expression.

Altogether, it suggests combined *Bifidobacterium longum* and active vitamin D has synergistic effects on the severity of *Salmonella* colitis by enhancing antibacterial and anti-inflammatory effects, as well as highlighting the possible role of VDR in the synergistic effects.

#### 3.1.3. Combination of 1,25D3 and Bifidobacterium Longum Exerted Reduction of Bacterial Translocation in Salmonella-Infected Mice

**Figure 3:**
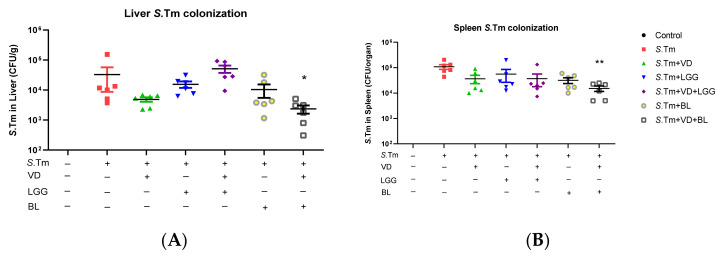
Combination of probiotics and active 1,25D3 attenuate systemic translocation of *Salmonella*. We infected 6–8 week-old female C57BL/6 mice with streptomycin, and incubated them with 10^8^ CPU of *S. typhimurium* (SL1344 strain) for 48 h, along with vehicle, 1,25D3 or probiotics, *Lactobacillus rhamnosus* GG (LGG) or *Bifidobacterium longum* (BL)*,* daily as shown above. Numbers of bacteria translocated to liver or spleen were measured from liver (**A**) or spleen (**B**) homogenates of infected and/or treated mice. The data shown are represented as the means ± the SEM of the bacterial load in the liver and spleen (*n* = 6). * *p* < 0.05, ** *p* < 0.01.

Khailova et al. [33] showed that 1,25D3 can reduce mortality and systemic bacterial translocation in experimental sepsis in weanling mice and decrease bacterial translocation into the liver and spleen in *Salmonella* colitis mice model [21]. To further determine the impact of the combination of probiotics and 1,25D3 treatment on tissue bacterial loads, liver and spleen were collected from *Salmonella*-infected mice treated with probiotics and/or active 1,25D3, homogenized and plated on LB plates. The CFU were determined. It was revealed that the combination of active 1,25D3 and probiotic *Bifidobacterium longum* treatment exerted reduction of bacterial loads in liver of *Salmonella*-infected mice (Figure 3).

Altogether, the above in vivo study demonstrated the combination of probiotic *Bifidobacterium longum* and active vitamin D3 reduced the severity of *Salmonella* colitis by their synergistic effects on the suppression of inflammatory responses, and blocking the translocation of bacteria by enhancing antimicrobial peptide.

### 3.2. In Vitro Study

#### 3.2.1. Combination of Bifidobacterium Longum and 1,25D3 Exerted Synergistic Effect on Vitamin D Receptor mRNA Expression in Salmonella-Infected SW480 Cells

In World J. Gastroenterol. [23], we demonstrated that active vitamin D enhanced VDR mRNA and protein expressions in *Salmonella*-infected intestinal epithelial cells (IECs). To investigate the regulatory effects of *Bifidobacterium longum* and active vitamin D on vitamin D receptor (VDR) mRNA expression in IECs infected by *Salmonella*, the cultured SW480 cells were infected by SL1344 after treatment with *Bifidobacterium longum*, 1,25D3 or combination of both. Total RNA was extracted and analyzed for VDR mRNA expression by real-time quantitative PCR.

**Figure 4:**
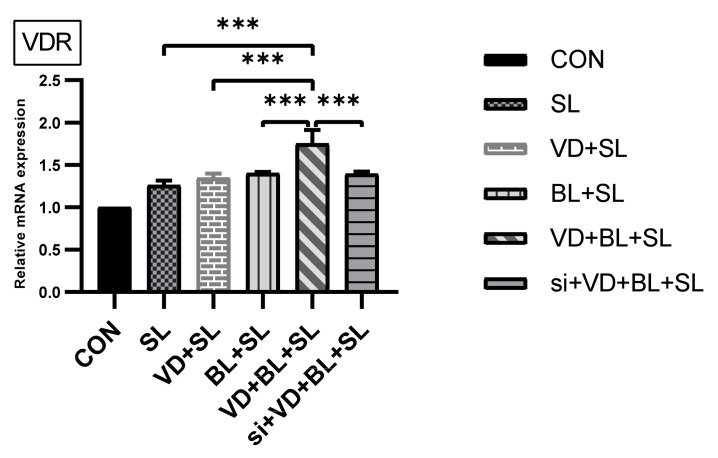
The synergistic effects of active vitamin D and *Bifidobacterium longum* on VDR mRNA expression in *Salmonella*-infected SW480 cells. SW480 cells were uninfected (CON) or infected by S. typhimurium wild-type strain SL1344 (SL) in a constant concentration, with and without treatment of *Bifidobacterium longum* (BL), 1,25D3 (VD) or combination of both (VD+BL). Transfection of SW480 cells with VDR siRNA (si) was performed to knockdown VDR Total RNA extracted from SW480 cells was prepared for analyzing the levels of VDR mRNA by real-time quantitative PCR. The results of mRNA levels were depicted as means ± SEM of at least three independent experiments. *** *p* < 0.001.

As shown in Figure 4, *Salmonella* infection induced VDR mRNA expression (normalized to GAPDH) in SW480 cells, which was synergistically enhanced by combination of *Bifidobacterium longum* and 1,25D3. Knockdown of VDR by siRNA counteracted the synergistic effect of the combination.

#### 3.2.2. The Synergistic Effect of Bifidobacterium Longum on Vitamin D-Induced mRNA Expression Is Dependent on VDR

**Figure 5:**
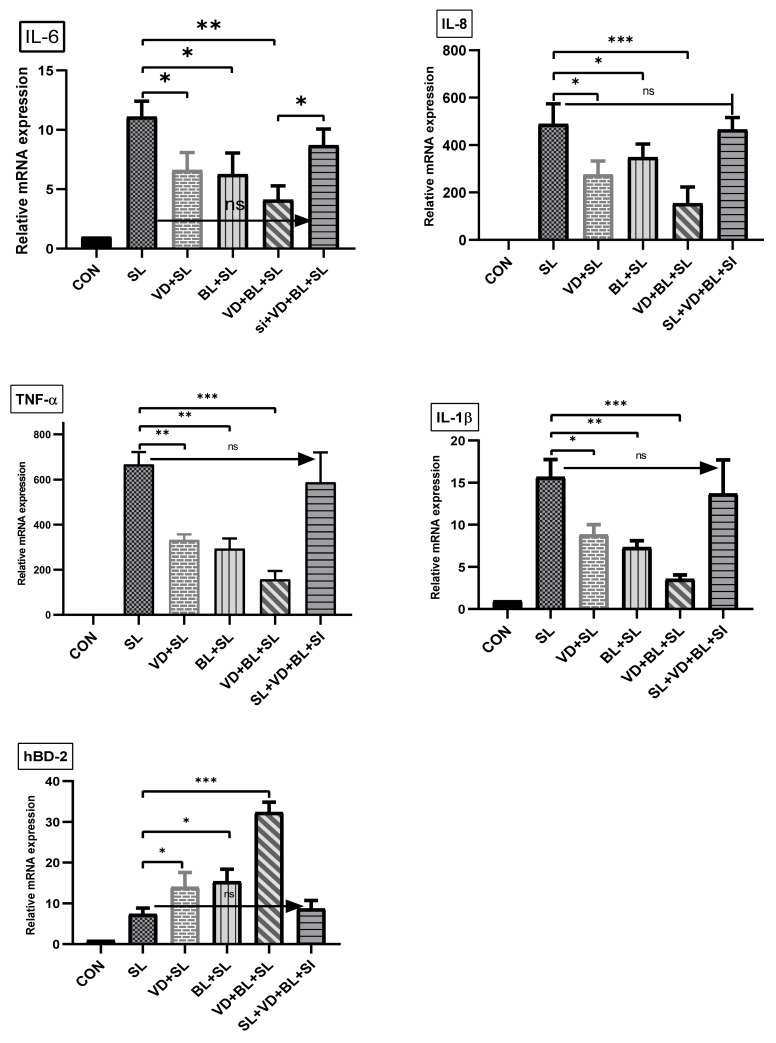
Involvement of VDR in synergistic effects of combined *Bifidobacterium longum* and active vitamin D3 on *Salmonella*-induced IL-8, IL-1β, TNF-α and hBD-2 mRNA expression in SW480 cells. Transfection of SW480 cells with VDR siRNA (si) was performed to knockdown VDR. Transfected SW480 cells were uninfected (CON) or infected by *S. typhimurium* wild-type strain SL1344 (SL) in a constant concentration, in the absence or presence of *Bifidobacterium longum* (BL), 1,25D3 (VD) or combination of both (VD+BL). Total RNA was extracted, reverse transcribed and real-time quantitative PCR was performed to estimate amounts of IL-6, IL-8, IL-1β, TNF-αand hBD-2transcript in *Salmonella*-infected cultured cells. Relative quantification based on internal reference gene (GAPDH transcript) to determine fold-differences in expression of the target genes, IL-8, IL-1β, TNF-α and hBD-2 mRNA, are measured and the fold increase over uninfected control cells is shown. Results are represented as mean ± SEM for at least three determinations from independent experiments. An asterisk indicates a significant difference (* *p* < 0.05, ** *p*< 0.01, *** *p* < 0.001, ns: not significant).

To further verify the role of VDR on the synergistic effects of *Bifidobacterium longum* on vitamin D-induced inflammatory responses and AMPs, we adapted a siRNA knock-down approach for VDR. Knockdown of VDR was confirmed by Western blot with specific siRNA in SW480 cells up to 48 h previously [23]. siRNA-transfected SW480 cells were untreated or treated with vitamin D3 alone or in combination with *Bifidobacterium longum* and vitamin D3 for 6 h. Following the knockdown of VDR, we detected that the synergistic effect of *Bifidobacterium longum* on vitamin D3 -induced IL-8, IL-1β, TNF-α and hBD-2 mRNA expression in SW480 cells was almost completely diminished in VDR-silenced cells (Figure 5), but not in control siRNA-silenced cells (data not shown). Therefore, specific suppression by siRNA targeting VDR diminished the synergistic effect of *Bifidobacterium longum* on vitamin D3 -induced IL-8, IL-1β, TNF-α and hBD-2 mRNA expression.

## 4. Discussion

It has been demonstrated [19] that 1,25D3 was effective in ameliorating DSS-induced acute colitis by enhancing epithelial cell resistance to injury and suppressing proinflammatory responses to luminal antigens. Based on our recent study demonstrating the benefit of active vitamin D on *Salmonella* colitis [21] and probiotics on gastroenteritis and *E. coli* infection [24,25], it is reasonable and promising to investigate the benefits of combining both supplements to treat *Salmonella* colitis. However, to our knowledge, there is no such report in the literature. Daily supplements of the probiotic strain *Lactobacillus reuteri* NCIMB 30242 increased the mean circulating vitamin D levels by 25% [34]. It suggests the synergistic benefits of probiotics and vitamin D to protect against a range of serious health problems and provide an effective treatment option for colitis. A systematic review of randomized controlled trials [35] revealed co-supplementation of vitamin D and probiotics generated greater health benefits than its comparators did in nearly all studies, including coronary heart disease, gestational diabetes, infantile colic, osteopenia, polycystic ovarian syndrome, schizophrenia, and type 2 diabetes. Our in vivo study demonstrated the combination of probiotic *BL* and active vitamin D3 had synergistic effects on reducing *Salmonella* colitis by the suppression of inflammatory responses, and blocking the translocation of bacteria.

Why are the synergistic effects of *BL* better than *LGG* when they are in combination with active vitamin D? One clear difference between *Lactobacillus* and *Bifidobacterium* is that *Bifidobacterium* reside mainly in the colon while *Lactobacillus* strains are mainly found in the small intestine. Although treatment with probiotics usually has a positive effect in irritable bowel syndrome (IBS), four clinical trials using different *Lactobacillus* strains failed to show any reduction in IBS symptoms over placebo [36]. *BL* was partly superior to other species of *Lactobacillus* for the treatment of post-infectious irritable bowel syndrome [36]. Moreover, seventy-seven subjects with IBS were randomized to receive either *Lactobacillus* or *Bifidobacterium* for 8 weeks [37]. *B. infantis* 35624 alleviates symptoms in IBS, superior to *Lactobacillus salivarius* UCC4331, which is similar to another study by O’Sullivan and O’Morain [38] who failed to detect an effect of *LGG* on overall symptomatology. *Bifidobacterium* is reported to be able to modify the gut microbiota by producing organic acids, such as butyrate acid, and competitively adhering to the mucosa and epithelium [39]; therefore, it has a great ability to colonize the intestine. By producing organic acids, *Bifidobacterium longum* ATCC 15707 also has the ability to repress *Clostridium difficile* infection [40] and reduce the severity of chemical colitis on rats [41]. The selection of strains from of the *L. rhamnosus* group is a special field of probiotic research [42]. Since *L. casei* and *L. rhamnosus* strains are transient organisms in the intestine, *BL* has been preferred because it has fewer demanding requirements for anaerobic conditions in foods and in pharmaceuticals. Therefore, our study suggests that specific strains of probiotics should be chosen according to the involved segment of intestine. For example, *Lactobacillus* should be used in diseases of the small intestine, while *Bifidobacterium* is beneficial for colonic diseases.

Our recent studies [22,23] observed that active vitamin D enhanced hBD-2 in *Salmonella*-infected IECs to protect the host against infection, while it downregulated proinflammatory responses (IL-8 & IL-1β) to prevent the host from the detrimental effects of overwhelming inflammation [22,23]. Probiotic bacteria are microorganisms that benefit the host through the improvement of the balance of intestinal microflora and possibly by augmentation of host defense systems. Lactic acid bacteria, *LGG* and *B. bifidum*, enhance autophagic ability of mononuclear phagocytes [43] as well as production of enterocyte β–defensin 2 via many signaling pathways [44,45]. Defensins disrupt the integrity of the bacterial cell membrane, resulting in the death of the microbe. Enteric defensins are essential regulators of homeostatic commensal microbiota [46]. Spontaneous colitis developing in mice with defective expression of defensins is associated with increased commensal bacterial translocation [47]. Additionally, our team observed that probiotics suppress IL-1beta expression in *Salmonella*-infected IECs [27]. Accordingly, we observed decreased *Salmonella* translocation in liver and spleen by probiotic BL or active vitamin D, significantly by the combination of both. We also demonstrated that the combination of probiotic and active vitamin D synergistically increase antimicrobial peptide hBD-2 but decrease inflammatory responses including IL-1β, Il-8, IL-6 and TNF-α. It suggests that combination of probiotic and active vitamin D may enhance bacterial killing by β-defensin and lessen detrimental inflammation-induced translocation of the bacteria.

VDR-defect mice are much more susceptible to mucosal injury, leading to extensive ulceration and early death [48]. VDR-defective mice were hyper-responsive to exogenously injected LPS. Moreover, chemical-induced colitis in the VDR knockout mice was accompanied by high colonic expression of inflammation, including TNF-α, IL-1α, IL-1β and IL-8, leading to weight loss, ulceration and perforation of the bowel, endotoxemia and increased mortality [49]. Our in vitro study observed that 1,25D3 reduces *Salmonella*-induced IL-8 and IL-1β but enhances VDR expression in IECs [22]. Besides 1,25D3 attenuates the severity of *Salmonella* colitis by downregulating inflammatory responses [21]. It suggests that VDR may play an anti-inflammatory role in *Salmonella* colitis. Oral supplementation of probiotics has been observed to increase serum levels of 25-hydroxyvitamin D in a human study [34]. Furthermore, probiotics can potentially affect the uptake of VDR by enterocytes [50], enhancing its expression and activity [51]. The colonization of commensal bacteria (or probiotics) in the intestine may orchestrate their interaction with VDR by affecting both the distribution and expression of VDR in IECs [52]. Probiotics could increase VDR signaling and inhibit infection-induced inflammation in both human and mouse IECs [53]. The authors conclude that the VDR pathway is required for probiotic protection in *Salmonella* colitis and also detected that probiotics increased the expression of the VDR target genes, such as antimicrobial peptides at the transcriptional level. We also demonstrated that probiotics exert anti-inflammatory (IL-8 and IL-1β) and antibacterial (hBD-2) effects in *Salmonella*-infected IECs [26] with the involvement of VDR [27]. Compatible with these findings, we demonstrated the combination of probiotic and active vitamin D synergistically enhanced VDR mRNA expression in *Salmonella* colitis mice, contributing to the synergistic effects of the combination on anti-inflammation and antimicrobial peptides by in vitro study. Thus, co-supplementation with probiotic and vitamin D3 could provide a new and promising strategy to reduce antibiotic use and hence prevent the emerging epidemic of bacterial resistance.

## 5. Conclusions

Our in vivo study demonstrated that the combination of probiotic *B. longum* strain ATCC15707 with active vitamin D3 has a synergistic effect on reducing the severity of *Salmonella* colitis by the suppression of inflammatory responses, and blocking the translocation of *Salmonella* through the enhancement of antimicrobial peptides, with the involvement of VDR by in vitro study, whereas the *L. rhamnosus* GG strain ATCC53103 does not. The combination of probiotics and vitamin D could be an alternative treatment for *Salmonella* colitis and lessen the overuse of antibiotics. However, specific strains should be investigated to see their effects on vitamin D-mediated innate immunity in different diseases.

## Data Availability

The data presented in this study are available on request from the corresponding author.
